# Supplementary material for: Immunoinformatic approach to design a multiepitope vaccine targeting non-mutational hotspot regions of structural and non-structural proteins of the SARS CoV2
Source: PeerJ. 2021 Mar 23;9:e11126. doi: 10.7717/peerj.11126 (PMC7996071; doi:10.7717/peerj.11126)
Supplement: Supplemental Information 1 [file peerj-09-11126-s001.pdf]

Supplementary Data

Title: Immunoinformatic approach to design a multiepitope vaccine targeting non-mutational hotspot regions of structural and non-structural proteins of the SARS CoV2.

Vandana Solanki, Monalisa Tiwari, and Vishvanath Tiwari*

Department of Biochemistry, Central University of Rajasthan, Bandarsindri, Ajmer-305817, India
*To whom correspondence should be addressed,
E-mail: vishvanath@curaj.ac.in
Mobile No.: + 91-850-300-2573

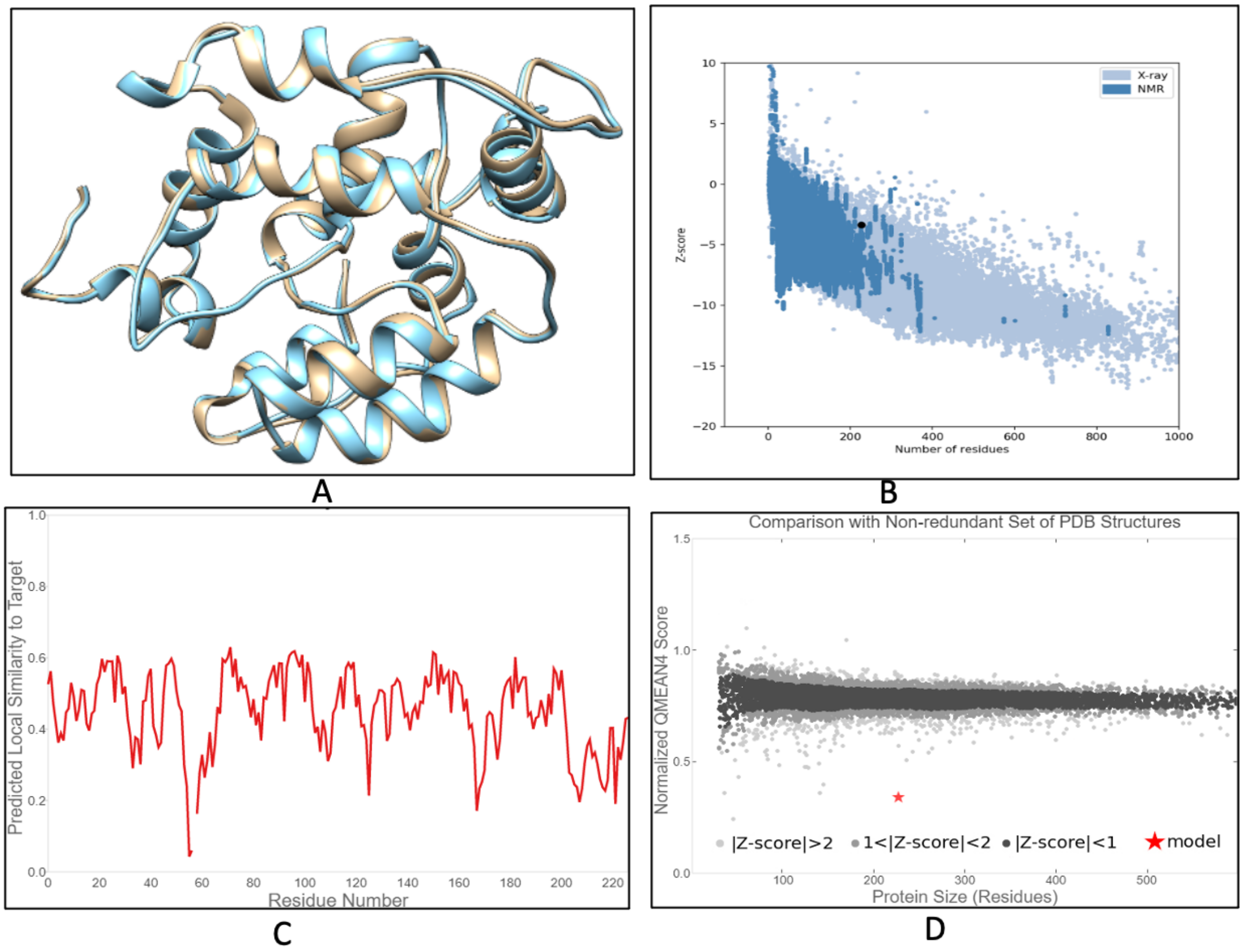

Supplementary Figure SF1: Comparative analysis of Phyre2 modelled VTC 3 construct structure with GalaxyRefined PDB where brown colour shows the VTC3 construct & blue colour represent the refined PDB that showed the 97% residue in allowed region (A). The overall quality score analysis by the ProSA (B) and QMEAN (C & D) server of the VTC 3 vaccine.

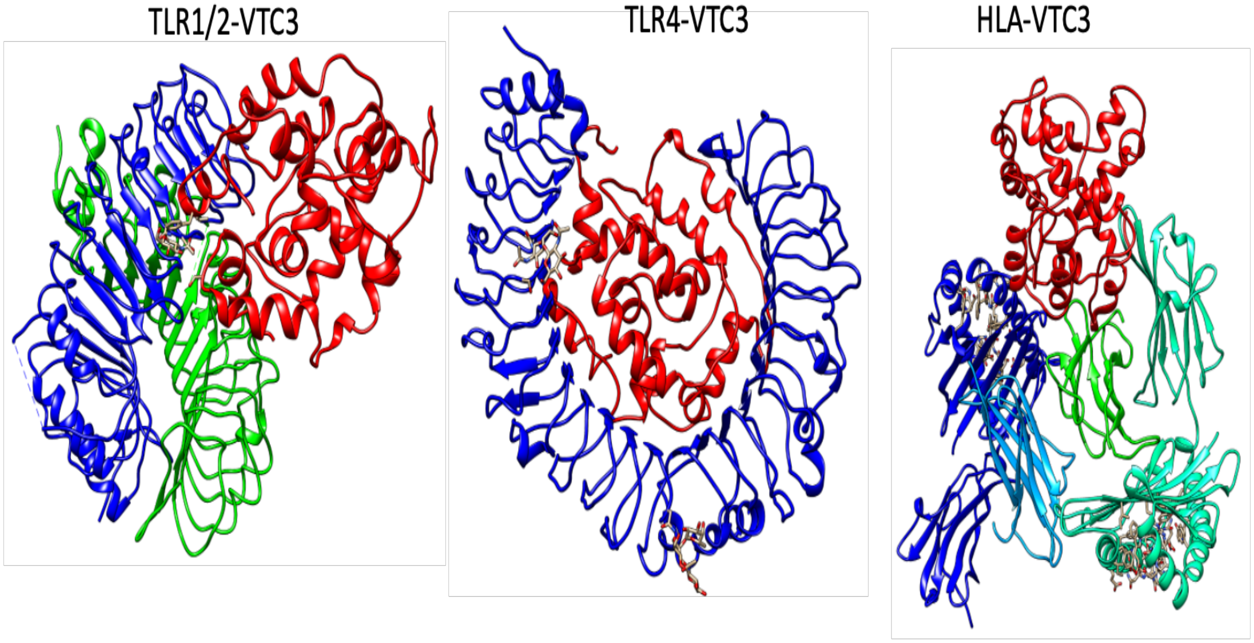

Supplementary Figure SF2: Representation of Docked TLR1/2, TLR4, & HLA alleles with VTC3 construct using HDOCK server: A) TLR1/2(Blue-Green)-VTC3(Red) complex showed the docking score of -260, B) TLR4(Blue)-VTC3(RED) complex docking score of -287 and C) HLA allele(Green-Blue)-VTC3(Red) showed the binding affinity of -233. These score represents the good binding affinities of TLRs-VTC3 & HLA-VTC3 complexes

Supplementary Table ST1: The calculated binding free energy (ΔG) and dissociation constant (K) for the docked VTC3-TLR1/2, VTC3-TLR4, and VTC3-HLA complex

S.No	Name	Patchdock			HDOCK		
		ΔG (kcal/mol)	K (M) at 25.0 °C	Docking score	ΔG (kcal/mol)	K (M) at 25.0 °C	Docking score
1	VTC3-TLR1/2	-11.9	$1.8x10^{-9}$	17536	-15.9	$2.1x10^{-12}$	-260
2	VTC3-TLR4	-8.5	$5.4x10^{-7}$	20820	-16.2	$1.2x10^{-12}$	-287
3	VTC3-HLA	-8.0	$1.5x10^{-6}$	20302	-12.6	$5.8x10^{-10}$	-233

Supplementary Table ST2: The calculated binding free energy and dissociation constant for the docked VTC3-HLA, VTC3-TLR1/2 & VTC3-TLR4 complex during different time periods of molecular dynamics simulation.

S.No	Name	Time	ΔG (kcal/mol)	K (M) at 25.0 °C
1	VTC3-HLA complex	1 ns	-8.4	6.6x10^{-7}
		5 ns	-8.2	9.6 x10^{-7}
		10 ns	-9.3	1.6 x10^{-7}
		15 ns	-9.8	6.1 x10^{-8}
		20 ns	-10.4	2.3 x10^{-8}
		25 ns	-10.8	1.2 x10^{-8}
		30 ns	-12.5	6.8 x10^{-10}
		35 ns	-13.1	2.3 x10^{-10}
		40 ns	-11.9	2 x10^{-9}
		45 ns	-13.1	2.4 x10^{-10}
		50 ns	-12.3	8.9 x10^{-10}
2	VTC3-TLR1/2 complex	1ns	-12.9	3.4x10-^{10}
		5ns	-12.8	3.8x10-^{10}
		10ns	-11.5	3.8x10^{-9}
3	VTC3-TLR4 complex	1ns	-7.8	1.8X10^{-6}
		5ns	-8.7	3.9x10^{-7}
		10ns	-9.1	2.1x10^{-7}

Supplementary Table ST3: Interacting residues of multi-epitope vaccine VTC3 with HLA complex at simulation time 50ns.

VTC3 amino acid	VTC3 residue Number	HLA amino acid	HLA residue	Chain number
TYR	158	GLU	74	D
GLY	167	ASN	17	D
CYS	7	HIS	188	C
PHE	42	GLY	18	D
PHE	42	ASN	17	D
MET	168*	ARG	97	D
ALA	2	GLU	16	D
LYS	5	HIS	13	D
CYS	7	HIS	191	C
ALA	166	GLU	74	D
PHE	37	LYS	19	D
ARG	169*	PRO	193	C
TYR	158	THR	73	D
GLU	1	GLU	16	D
CYS	9	HIS	277	C
CYS	9	TRP	274	C
CYS	7	TRP	274	C
ALA	166	ALA	15	D
ARG	165	ALA	15	D

ASP	10	HIS	277	C
GLU	14	HIS	277	C
GLY	167	ARG	97	D
ARG	165	LYS	19	D
CYS	7	VAL	189	C
CYS	7	THR	190	C
PHE	37	GLY	18	D
LYS	5	HIS	188	C
CYS	9	PRO	276	C
GLU	1	LYS	19	D
LEU	17	HIS	277	C
ALA	166	GLU	16	D
SER	8	HIS	277	C
PHE	37	ASN	17	D
MET	168*	THR	190	C
ARG	13	HIS	277	C
ARG	165	GLU	16	D
SER	8	TRP	274	C
CYS	9	GLU	275	C
PHE	42	THR	73	D
GLU	157	GLU	74	D
ALA	166	ARG	97	D
LYS	5	GLU	16	D
SER	8	GLU	275	C
TYR	158	ASP	76	D
TYR	158	LYS	75	D
ALA	166	ASN	17	D
PHE	42	PRO	72	D
ASP	41	GLY	18	D
MET	168*	GLU	74	D
LYS	5	PRO	14	D
ARG	165	ASN	17	D
GLU	157	ASN	17	D
GLY	167	GLU	74	D

***Showed single mutation**

Supplementary Table ST4: Identification of similarity between VTC3 construct and protein of organism form gut microbiome.

S.No.	Organism Names	E-value & Percent Identity
1.	Erysipelatoclostridium ramosum DSM 1402	No
2.	Lactobacillus ruminis ATCC 25644	No
3.	Clostridium methylpentosum DSM 5476	No
4.	Staphylococcus sp. HGB0015	No
5.	Eggerthella lenta 1_1_60AFAA	E value 1, Identity 42.7 %
6.	Coprococcus eutactus ATCC 27759	No
7.	Streptomyces sp. HGB0020	No
8.	Parabacteroides goldsteinii DSM 19448 = WAL 12034	E value 0.54, Identity 31.15 %
9.	Corynebacterium sp. HFH0082	No
10.	Eubacterium sp. 3_1_31	No

11.	Clostridium perfringens WAL-14572	No
12.	Proteus penneri ATCC 35198	No
13.	Prevotella salivae DSM 15606	No
14.	Blautia obeum ATCC 29174	No
15.	Ruminococcus sp. 5_1_39BFAA	E value 4.3, Identity 48.00 %
16.	Erysipelotrichaceae bacterium 2_2_44A	No
17.	Ruminococcus gnavus ATCC 29149	No
18.	Anaerostipes caccae DSM 14662	No
19.	Parvimonas micra ATCC 33270	No
20.	Alistipes putredinis DSM 17216	No
21.	Bacteroides cellulosilyticus DSM 14838	E value 2.3, Identity 36.67%
22.	Edwardsiella tarda ATCC 23685	No
23.	Helicobacter pullorum MIT 98-5489	No
24.	Bacteroides pectinophilus ATCC 43243	No
25.	Bacteroides caccae ATCC 43185	No
26.	Helicobacter bilis ATCC 43879	No
27.	Faecalibacterium prausnitzii M21/2	E value 4.6 , Identity 32.73 %
28.	Lactobacillus brevis subsp. gravesensis ATCC 27305	No
29.	Lactobacillus rhamnosus LMS2-1	E value 1.7, Identity 36.71 %
30.	Bacteroides fluxus YIT 12057	E value 2.7, Identity 33.33 %
31.	Bifidobacterium dentium ATCC 27678	No
32.	Enterobacteriaceae bacterium 9_2_54FAA	No
33.	Dialister succinatiphilus YIT 11850	E value 5.2, Identity 23.93 %
34.	Veillonella sp. 6_1_27	No
35.	Bacillus smithii 7_3_47FAA	No
36.	Campylobacter coli JV20	No
37.	Bilophila wadsworthia 3_1_6	No
38.	Parabacteroides johnsonii DSM 18315	E value 7, Identity 36.10 %
39.	Helicobacter canadensis MIT 98-5491	No
40.	Desulfovibrio piger ATCC 29098	No
41.	Dysgonomonas gadei ATCC BAA-286	No
42.	Anaerobaculum hydrogeniformans ATCC BAA-1850	No
43.	Anaerostipes hadrus DSM 3319	E value 2.3, Identity 43.59 %
44.	Escherichia coli MS 85-1	No
45.	Leuconostoc mesenteroides subsp. cremoris ATCC 19254	No
46.	Phascolarctobacterium succinatutens YIT 12067	No
47.	Clostridium bolteae ATCC BAA-613	No
48.	Turicibacter sp. HGF1	No
49.	Collinsella intestinalis DSM 13280	No
50.	Bacteroides xylanisolvens SD CC 1b	No
51.	Bacteroides intestinalis DSM 17393	E value 2, Identity 36.67%
52.	Catenibacterium mitsuokai DSM 15897	No
53.	Dorea sp. D27	E value 1.4, Identity 36.84 %
54.	Parabacteroides sp. HGS0025	No
55.	Bifidobacterium breve DSM 20213 = JCM 1192	E value 4.5, Identity 22.99 %
56.	Streptococcus anginosus 1_2_62CV	No
57.	Clostridium spiroforme DSM 1552	No
58.	Helicobacter cinaedi CCUG 18818 = ATCC BAA-847	No
59.	Subdoligranulum sp. 4_3_54A2FAA	E value 2.6, Identity 52.36 %

60.	Citrobacter youngae ATCC 29220	No
61.	Campylobacter sp. 10_1_50	No
62.	Bifidobacterium sp. 12_1_47BFAA	No
63.	Sutterella parvirubra YIT 11816	No
64.	Bacteroides stercoris ATCC 43183	E value 2.3, Identity 33.33 %
65.	Ruminococcus callidus ATCC 27760	No
66.	Desulfovibrio sp. 6_1_46AFAA	No
67.	Eubacterium siraeum DSM 15702	E value 7.9, Identity 35.71 %
68.	Holdemanella biformis DSM 3989	No
69.	Providencia alcalifaciens F90-2004	No
70.	Ralstonia sp. 5_2_56FAA	No
71.	Bacteroides fragilis 3_1_12	No
72.	Dermabacter sp. HFH0086	No
73.	Eubacterium hallii DSM 3353	No
74.	Enterococcus saccharolyticus 30_1	No
75.	Paenibacillus sp. HGF5	No
76.	Blautia hansenii DSM 20583	No
77.	Helicobacter macacae MIT 99-5501	No
78.	Campylobacter upsaliensis JV21	No
79.	Synergistes sp. 3_1_syn1	No
80.	Coprococcus comes ATCC 27758	No
81.	Bacteroides clarus YIT 12056	E value 2.8, Identity 33.33 %
82.	Clostridium symbiosum ATCC 14940	E value 2.6, Identity 43.24 %
83.	Collinsella aerofaciens ATCC 25986	No
84.	Lactobacillus amylolyticus DSM 11664	No
85.	Dorea formicigenerans 4_6_53AFAA	
86.	Bacteroides plebeius DSM 17135	E value 0.071, Identity 31.94 %
87.	Eubacterium ventriosum ATCC 27560	
88.	Fusobacterium nucleatum subsp. animalis ATCC 51191	E value 0.50, Identity 26.67 %
89.	Providencia rettgeri DSM 1131	No
90.	Prevotella oralis HGA0225	No
91.	Lactobacillus ultunensis DSM 16047	No
92.	Prevotella copri DSM 18205	No
93.	Fusobacterium necrophorum subsp. funduliforme 1_1_36S	E value 0.81, Identity 34.36 %
94.	Clostridium hylemonae DSM 15053	E value 2.8, Identity 45.71 %
95.	Bacteroides coprophilus DSM 18228 = JCM 13818	No
96.	Methanobrevibacter smithii DSM 2375	No
97.	Clostridium] leptum DSM 753	E value 3.3, Identity 66.67 %
98.	Anaerofustis stercorihominis DSM 17244	No
99.	Dysgonomonas mossii DSM 22836	No
100.	Providencia rustigianii DSM 4541	No
101.	Lactobacillus hilgardii DSM 20176 = ATCC 8290	No
102.	Tannerella sp. 6_1_58FAA_CT1	No
103.	Helicobacter winghamensis ATCC BAA-430	No
104.	Collinsella tanakaei YIT 12063	E value 0.015, Identity 47.06%
105.	Subdoligranulum variabile DSM 15176	No
106.	Odoribacter laneus YIT 12061	E value 6.9, Identity 38.46 %
107.	Collinsella stercoris DSM 13279	No
108.	Streptococcus equinus ATCC 9812	No

109.	Clostridium citroniae WAL-17108	E value 1.3, Identity 32.26 %
110.	Clostridium clostridioforme 2_1_49FAA	No
111.	Pseudomonas sp. 2_1_26	E value 0.74, Identity 31.03 %
112.	Proteus mirabilis WGLW6	No
113.	Flavonifractor plautii ATCC 29863	E value 5.4, Identity 55.56 %
114.	Bifidobacterium adolescentis L2-32	No
115.	Propionibacterium sp. HGH0353	E value 8.1, Identity 24.00 %
116.	Clostridium scindens ATCC 35704	No
117.	Enterococcus faecalis 918	No
118.	Paraprevotella clara YIT 11840	No
119.	Lactobacillus plantarum subsp. plantarum ATCC 14917 = JCM 1149 = CGMCC 1.2437	E value 0.65, Identity 21.43 %
120.	Paraprevotella xylaniphila YIT 11841	No
121.	Listeria grayi DSM 20601	No
122.	Anaerotruncus colihominis DSM 17241	No
123.	Eggerthella sp. HGA1	No
124.	Bacillus sp. 7_6_55CFAA_CT2	No
125.	Eubacterium cylindroides ATCC 27803	No
126.	Coprococcus sp. HPP0074	E value 1.8, Identity 75.00 %
127.	Slackia piriformis YIT 12062	No
128.	Bifidobacterium gallicum DSM 20093 = LMG 11596	No
129.	Bifidobacterium pseudocatenulatum DSM 20438 = JCM 1200 = LMG 10505	E value 9.8, Identity 42.86 %
130.	Lactobacillus helveticus DSM 20075 = CGMCC 1.1877	No
131.	Streptococcus infantarius subsp. infantarius ATCC BAA-102	No
132.	Collinsella sp. 4_8_47FAA	No
133.	Alistipes indistinctus YIT 12060	No
134.	Bacteroides uniformis ATCC 8492	E value 2.1, Identity 27.12 %
135.	Clostridium orbiscindens 1_3_50AFAA	No
136.	Intestinibacter bartlettii DSM 16795	E value 0.013, Identity 28.81 %
137.	Butyricicoccus pullicaecorum 1.2	No
138.	Barnesiella intestinihominis YIT 11860	No
139.	Oscillibacter sp. KLE 1745	No
140.	Acinetobacter junii SH205	No
141.	Listeria innocua ATCC 33091	No
142.	Clostridium asparagiforme DSM 15981	No
143.	Bilophila sp. 4_1_30	No
144.	Corynebacterium ammoniagenes DSM 20306	No
145.	Enterobacter cancerogenus ATCC 35316	No
146.	Lachnospiraceae bacterium 3_1_57FAA_CT1	No
147.	Bacteroides coprocola DSM 17136	No
148.	Eubacterium ramulus ATCC 29099	No
149.	Coprobacillus sp. 3_3_56FAA	No
150.	Bacteroides sp. 224	E value 9.3, Identity 25.19 %
151.	Pediococcus acidilactici 7_4	No
152.	Dorea longicatena DSM 13814	No
153.	Aneurinibacillus aneurinilyticus ATCC 12856	No
154.	Cetobacterium somerae ATCC BAA-474	No
155.	Butyrivibrio crossotus DSM 2876	No

156.	Oxalobacter formigenes HOxBLS	No
157.	Lactobacillus buchneri ATCC 11577	No
158.	Pseudoflavonifractor capillosus ATCC 29799	E value 6, Identity 55.56 %
159.	Bacteroides eggerthii 1_2_48FAA	E value 0.071, Identity 28.81 %
160.	Blautia hydrogenotrophica DSM 10507	No
161.	Fusobacterium varium ATCC 27725	No
162.	Lactobacillus fermentum ATCC 14931	E value 5, Identity 64.29 %
163.	Roseburia intestinalis L1-82	No
164.	Streptococcus sp. 2_1_36FAA	E value 1.9, Identity 30.00 %
165.	Clostridium celatum DSM 1785	No
166.	Ruminococcaceae bacterium D16	E value 5, Identity 55.56 %
167.	Bifidobacterium bifidum ATCC 29521 = JCM 1255 = DSM 20456	No
168.	Peptoclostridium difficile NAP08	No
169.	Mitsuokella multacida DSM 20544	No
170.	Ruminococcus lactaris ATCC 29176	No
171.	Ruminococcus torques ATCC 27756	No
172.	Clostridium sporogenes ATCC 15579	E value 7.9, Identity 42.11 %
173.	Succinatimonas hippei YIT 12066	No
174.	Holdemania filiformis DSM 12042	No
175.	Bacteroides oleiciplenus YIT 12058	E value 2.3, Identity 36.67 %
176.	Lactobacillus delbrueckii subsp. lactis DSM 20072	No
177.	Yokenella regensburgei ATCC 43003	No
178.	Bifidobacterium angulatum DSM 20098 = JCM 7096	No
179.	Tyzzerella nexilis DSM 1787	No
180.	Porphyromonas sp. 31_2	E value 9.9, Identity 58.33 %
181.	Roseburia inulinivorans DSM 16841	No
182.	Bifidobacterium catenulatum DSM 16992 = JCM 1194 = LMG 11043	No
183.	Escherichia sp. 3_2_53FAA	E value 1.8, Identity 54.29 %
184.	Parasutterella excrementihominis YIT 11859	E value 8.8, Identity 57.89 %
185.	Arcobacter butzleri JV22	E value 0.66, Identity 37.50 %
186.	Helicobacter pylori GAM115Ai	No
187.	Bacteroides salyersiae WAL 10018 = DSM 18765 = JCM 12988	No
188.	Clostridiales bacterium 1_7_47FAA	E value 7.2, Identity 42.11 %
189.	Clostridium sp. KLE 1755	E value 4, Identity 35.71 %
190.	Bacteroides dorei DSM 17855	No
191.	Cedecea davisae DSM 4568	No
192.	Clostridium] hiranonis DSM 13275	No
193.	Hafnia alvei ATCC 51873	E value 1.7, Identity 55.56 %
194.	Weissella paramesenteroides ATCC 33313	No
195.	Prevotella stercorea DSM 18206	No
196.	Eubacterium] dolichum DSM 3991	No
197.	Acidaminococcus sp. D21	No
198.	Coprobacillus sp. D7	No
199.	Anaerococcus hydrogenalis DSM 7454	No
200.	Lactobacillus paracasei subsp. paracasei ATCC 25302	E value 8.6, Identity 38.24 %

201.	Clostridium] hathewayi DSM 13479	No
202.	Citrobacter sp. 30_2	No
203.	Megamonas funiformis YIT 11815	No
204.	Paenisporosarcina sp. HGH0030	No
205.	Blautia sp. KLE 1732	No
206.	Bacteroides vulgatus PC510	No
207.	Fusobacterium mortiferum ATCC 9817	E value 7.3, Identity 47.62 %
208.	Marvinbryantia formatexigens DSM 14469	No
209.	Fusobacterium ulcerans ATCC 49185	No
210.	Neisseria macacae ATCC 33926	E value 0.33, Identity 24.51 %
211.	Lactobacillus antri DSM 16041	E value 1.1, Identity 24.00 %
212.	Lactobacillus sp. 7_1_47FAA	No
213.	Helicobacter canis NCTC 12740	No
214.	Lactobacillus acidophilus ATCC 4796	No
215.	Parabacteroides merdae ATCC 43184	E value 2.4, Identity 42.86 %
216.	Desulfitobacterium hafniense DP7	No
217.	Acinetobacter radioresistens SH164	E value 1.4, Identity 31.25 %
218.	Burkholderiales bacterium 1_1_47	E value 9.5, Identity 57.89 %
219.	Citrobacter freundii 4_7_47CFAA	No
220.	Bacteroides ovatus 3_8_47FAA	No
221.	Bifidobacterium longum subsp. longum 2-2B	No
222.	Fusobacterium gonidiaformans ATCC 25563	E value 0.69, Identity 34.38 %
223.	Bacteroides finegoldii DSM 17565	No
224.	Sutterella wadsworthensis 3_1_45B	No
225.	Actinomyces sp. HPA0247	No
226.	Providencia stuartii ATCC 25827	No

Supplementary Table ST5: Table showing mutations sites in the epitopes of protein used for the multiepitope vaccine design.

Epitope	Protein	Vaccine	Mutations in amino acid positions of proteins
Epitope 1	ORF1ab Polyprotien (4380-4398)	6 to 24	No Mutation
Epitope 2	ORF1ab Polyprotien (3715-3723)	37-45	No Mutation
Epitope 3	ORF1ab Polyprotien (4933-4947)	51-65	No Mutation
Epitope 4	Surface Glycoprotein (406-421)	E78	E406Q
		R80	R408A, R408I
		Q81	Q409E
		A83	A411S
		G85	G413V
		Q86	Q414E, Q414K, Q414R
		T87	T415I
		G88	G416R
		K89	K417N, K417R

Epitope 5	Surface Glycoprotein (513-527)	S100	S514F
		E102	E516Q, E516V
		H105	H519P, H519Q, H519Y
		A106	A520S, A520V
		P107	P521R, P521S
		A108	A522E, A522P, A522S, A522V
		T109	T523A, T523N
		C111	C525F, C525W
		G112	G526V
Epitope 6	Surface Glycoprotein (258-266)	W126	W258C, W258L
		A128	A260S, A260T
		G129	G261C, G261D,G261R,G261V
		A130	A262D, A262G, A262S, A262T, A262V
		A131	A263E, A263S
		A132	A264V
		Y133	Y265C, Y265F, Y265N
		Y134	Y266C
Epitope 7	ORF3a (15-30)	L140	L15F, L15S
		K141	K16E, K16L, K16N
		Q142	Q17E, Q17H
		G143	G18V
		E144	E19A, E19D, E19K
		I145	I20T
		K146	K21E, K21N, K21R
		D147	D22E, D22G, D22Y
		A148	A23S, A23V
		T149	T24I, T24N
		P150	P25L, P25R, P25S
		S151	S26L, S26P
		D152	D27A, D27H, D27Y
		F153	F28C
		V154	V29F
		R155	R30H
Epitope 8	ORF3a (4-13)	M168	M5I
		R169	R6S
		I170	I7T
		F171	F8L
		T172	T9I
		T172	T9K
		G174	G11A
		T175	T12I
		V176	V13A, V13I, V13L
Epitope 9		Y182	Y59H

	Envelope protien (59-71)	R184	R61H, R61L
		V185	V62F
		N187	N64S
		L188	L65M
		N189	N66H
		S190	S68C, S68Y
		R192	R69I
		V193	V70F
		P194	P71L
Epitope 10	ORF8 protein (30-45)	P207	P30L, P30Q, P30S
		Y208	Y31H
		V209	V32G, V32I, V32L
		V210	V33A, V33I
		D211	D34E, D34V
		D212	D35E, D35Y
		P213	P36S
		C214	C37F
		P215	P38L, P38S, P38V
		I216	I39V
		H217	H40P, H40Q, H40Y
		F218	F41Y
		S220	S43F
		W222	W45A, W45C, W45L, W45S
